# EBNA1 binding and epigenetic regulation of gastrokine tumor suppressor genes in gastric carcinoma cells

**DOI:** 10.1186/1743-422X-11-12

**Published:** 2014-01-24

**Authors:** Fang Lu, Italo Tempera, Hyunna T Lee, Karen DeWispelaere, Paul M Lieberman

**Affiliations:** 1 The Wistar Institute, 3601 Spruce Street, Philadelphia, PA 19104, USA; 2 Department of Microbiology, The Fels Cancer Institute, Temple University School of Medicine, Philadelphia, PA, USA

**Keywords:** EBV, EBNA1, Gastric carcinoma, Gastrokine, ChIP-Seq, Epigenetic

## Abstract

**Background:**

Epstein-Barr Virus (EBV) latently infects ~10% of gastric carcinomas (GC). Epstein-Barr Nuclear Antigen 1 (EBNA1) is expressed in EBV-associated GC, and can bind host DNA, where it may impact cellular gene regulation. Here, we show that EBNA1 binds directly to DNA upstream of the divergently transcribed GC-specific tumor suppressor genes gastrokine 1 (GKN1) and gastrokine 2 (GKN2).

**Methods:**

We use ChIP-Seq, ChIP-qPCR, and EMSA to demonstrate that EBNA1 binds directly to the GKN1 and GKN2 promoter locus. We generate AGS-EBV, and AGS-EBNA1 cell lines to study the effects of EBNA1 on GKN1 and GKN2 mRNA expression with or without 5′ azacytidine treatment.

**Results:**

We show that gastrokine genes are transcriptionally silenced by DNA methylation. We also show that latent EBV infection further reduces GKN1 and GKN2 expression in AGS gastric carcinoma cells, and that siRNA depletion of EBNA1 partially alleviates this repression. However, ectopic expression of EBNA1 slightly increased GKN1 and GKN2 basal mRNA levels, but reduced their responsiveness to demethylating agent.

**Conclusions:**

These findings demonstrate that EBNA1 binds to the divergent promoter of the GKN1 and GKN2 genes in GC cells, and suggest that EBNA1 contributes to the complex transcriptional and epigenetic deregulation of the GKN1 and GKN2 tumor suppressor genes in EBV positive GC.

## Introduction

Epstein-Barr virus (EBV) is a human gammaherpesvirus found in a wide range of lymphoid and epithelial cell malignancies, including Burkitt’s lymphoma, Hodgkin’s disease, nasopharyngeal carcinoma (NPC), and post-transplant lymphoproliferative disease (reviewed in [1,2]). More recently, EBV has been found in ~10% of all gastric carcinoma (GC) cases worldwide [3,4]. EBV-associated GC has been shown to be a monoclonal outgrowth of EBV-infected gastric epithelial cells and is considered to be a distinct subtype of GC [5,6]. Because the incidence of GC is close to 900,000 people per year [7], EBV-associated GC may be among the most prevalent EBV-associated cancers.

In EBV positive gastric carcinoma cells, EBV establishes a variant type I latency, where EBV transcription is limited to the canonical type I genes EBNA1, EBERs, BART family non-coding RNA and miRNAs, but with some additional expression of LMP2A [6,8-11]. Among these latency genes, EBNA1 is the only viral nuclear protein that is detected in EBV-associated GC. EBNA1 is required for the establishment of the latent episomal infection and for the long-term survival of latently infected cells [12-15]. EBNA1 is a DNA binding protein that binds to both viral and host chromosomal sites. The binding sites in the viral genome have been characterized for essential functions in replication and transcriptional control of viral gene expression. However, the function of EBNA1 sequence-specific binding to the host chromosome is less well understood. While EBNA1 can bind to the promoter regions of several host genes, it remains unclear whether these genes are subject to EBNA1 regulation [12,16,17]. Overexpression of the EBNA1 DNA binding domain, which functions as a dominant negative in EBV infected cells, can inhibit cell viability in uninfected cells, suggesting that EBNA1 binds to and regulates cellular genes important for cell survival [18]. Ectopic expression of EBNA1 has been shown to effect host cell mRNA expression [19], but it is not clear whether these effects are direct or indirectly related to specific EBNA1 binding sites in the cellular genome.

In a previous study, we used ChIP-seq methods to analyze the genome-wide enrichment sites of EBNA1 in latently infected Raji Burkitt’s lymphoma cells and identified numerous cellular sites bound by EBNA1 [17]. Among those EBNA1 cellular enrichment sites we identified a significant EBNA1 binding peak located at the gastrokine 1 (GKN1) and gastrokine 2 (GKN2, also known as trefoil factor interacting protein (TFIZ1)) gene cluster. GKN1 and GKN2 have been identified based on their frequent loss of expression in neoplastic gastric carcinoma epithelial cells, compared to normal gastric tissue [20-22] (reviewed in [23]). Several recent studies have described anti-proliferative and anti-invasive activity for GKN1 in gastric epithelial cells, which, together with its frequent expression loss in cancer, suggests it functions as tumor suppressor specific to gastric epithelium [21,24-28]. GKN1 can inhibit cell migration and invasion in wound healing, transwell and Matrigel assay, as well as alter cell markers associated with the epithelial-mesenchymal transition [26]. GKN1 and GKN2 genes are located in close proximity and transcribed in opposite directions, suggesting that they likely share a bi-directional promoter, and are subject to coordinate regulation by shared transcription regulatory factors (reviewed in [23]).

In this study, we demonstrated the direct binding between EBNA1 and GKN1-GKN2 loci and investigated GKN1 and GKN2 gene expression modulation by EBV infection and EBNA1 protein. Our findings suggest that EBV infection can further inhibit GKN1 and GKN2 expression, and that loss of EBNA1 can facilitate epigenetic de-repression of GKN2 transcription. We also observed elevated DNA methylation levels at GKN1 and GKN2 promoter regions, and a potential role for EBNA1 in the deregulation and epigenetic repression of this tumor suppressor locus.

## Results

### Identification of a high-occupancy EBNA1 binding site at GKN1-GKN2 locus by ChIP-Seq

**Figure 1:**
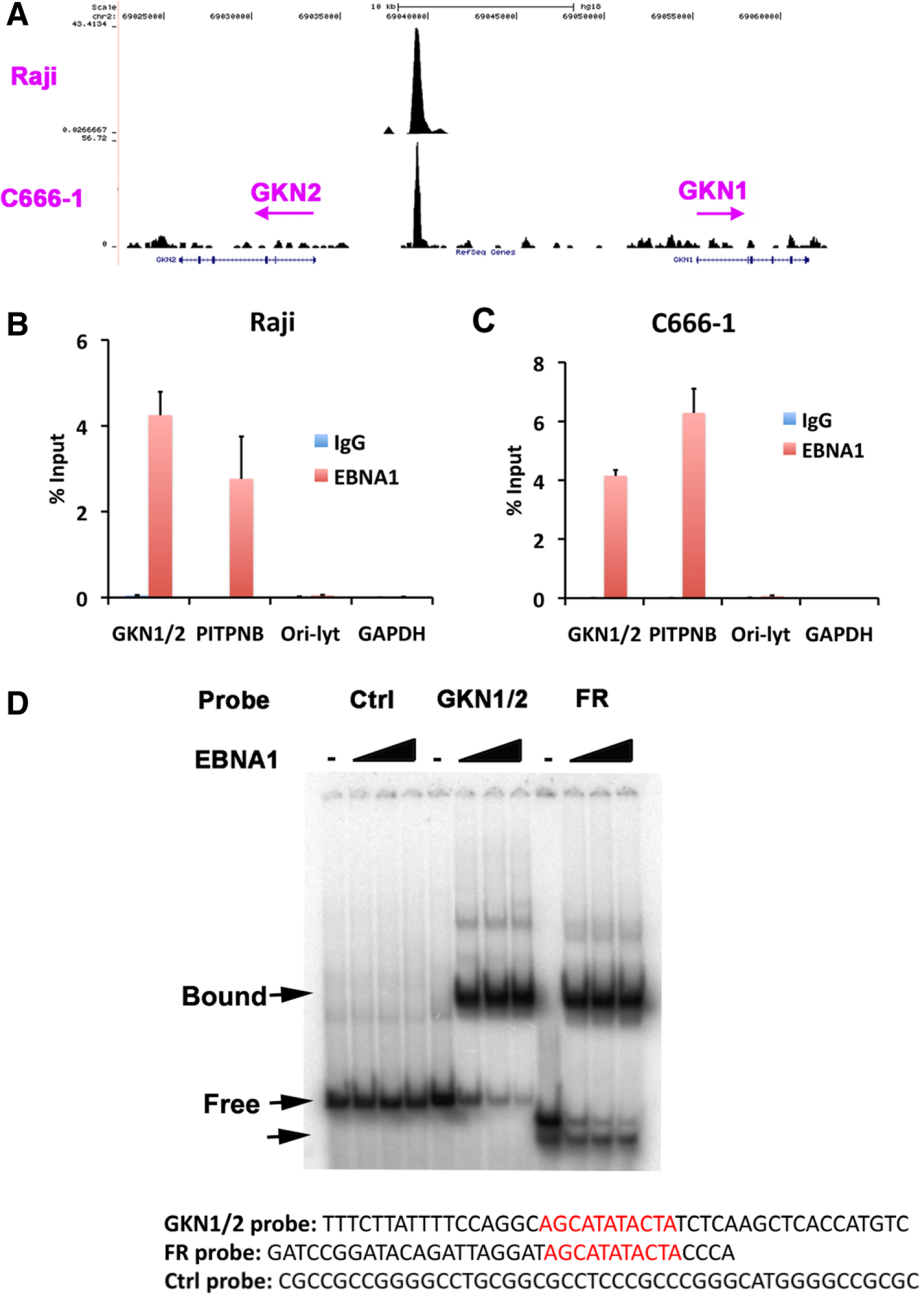
**EBNA1 binds at the GKN1 and GKN2 promoter locus. (A)** The UCSC genome browser was used to map EBNA1 binding peak generated from Raji and C666-1 ChIP-seq at the GKN1 and GKN2 gene loci. RefSeq annotated transcripts are indicated below the ChIP-Seq peak. **(B-C)** Realtime-PCR validation of ChIP-seq data for EBNA1 binding site at the GKN1/2 shared promoter region: EBNA1 (red bars) or control IgG (blue bars) were assayed by ChIP in Raji **(B)** or C666-1 cells **(C)** for DNA binding at the GKN1/2 site, PITPNB promoter, GAPDH, or EBV Ori-Lyt. **(D)** EMSA analysis of ^32^P labeled probes containing Control, GKN1/2 site, or EBV FR. Varying amount of EBNA1 DBD protein was used in binding reaction (0, 100, 300, 900 ng). Arrowheads represent EBNA1-specific bound complexes or free probe as indicated. The probe sequence of GKN1/2 site and FR is indicated with sequence homolog (red letters) between GKN1/2 and FR. The negative control (Ctrl) sequence is also indicated. Error bars indicate standard deviation from the mean (sdm) for n = 3.

Previously published ChIP-Seq data from Raji BL cells revealed a limited number of highly enriched EBNA1 binding sites based on peak scores and read numbers [17]. Further inspection revealed a strong EBNA1 binding site located upstream of the start sites for the divergently transcribed GKN1 and GKN2 genes (Figure 1A, upper track). A similar EBNA1 binding peak was observed in a separate ChIP-Seq experiment performed in EBV positive nasopharyngeal carcinoma cell line C666-1 (full ChIP-seq data to be published elsewhere), indicating that this binding occurs in both epithelial, as well as lymphoid cell types (Figure 1A, lower track). The center of the peak was located ~ 5 kb from GKN2 and ~15 kb from the GKN1 transcription start sites. ChIP-qPCR was used to validate the EBNA1 binding at the GKN1-GKN2 promoter region in both Raji and C666-1 cells (Figure 1B and C). qPCR indicated that EBNA1 bound to the GKN1-2 site with similar efficiency to a previously validated EBNA1-binding site at the PITPNB promoter. qPCR also indicated that EBNA1 binding to GKN1-GKN2 was specific since there was no detectable EBNA1 binding at either the cellular GAPDH locus or EBV OriLyt control regions, as expected (Figure 1B and C). The putative EBNA1 binding site at GKN1-GKN2 locus was identified by alignment with a consensus binding site in the EBV family of repeats (FR) region, and this sequence was then tested for direct binding to EBNA1 by EMSA (Figure 1D). Purified recombinant EBNA1 DBD protein was assayed for binding to probes containing the GKN1-GKN2 site (GKN1/2), FR, or a negative control sequence lacking a consensus EBNA1 binding site. We found that EBNA1 DBD bound efficiently to the GKN1-GKN2 site, as well as to the FR consensus, but did not bind to the control sequence. These findings suggest that EBNA1 interacts with GKN1-GKN2 promoter region through direct DNA-binding with the EBNA1 DBD.

### GKN1 and GKN2 mRNA are highly expressed in primary stomach tissue

**Figure 2:**
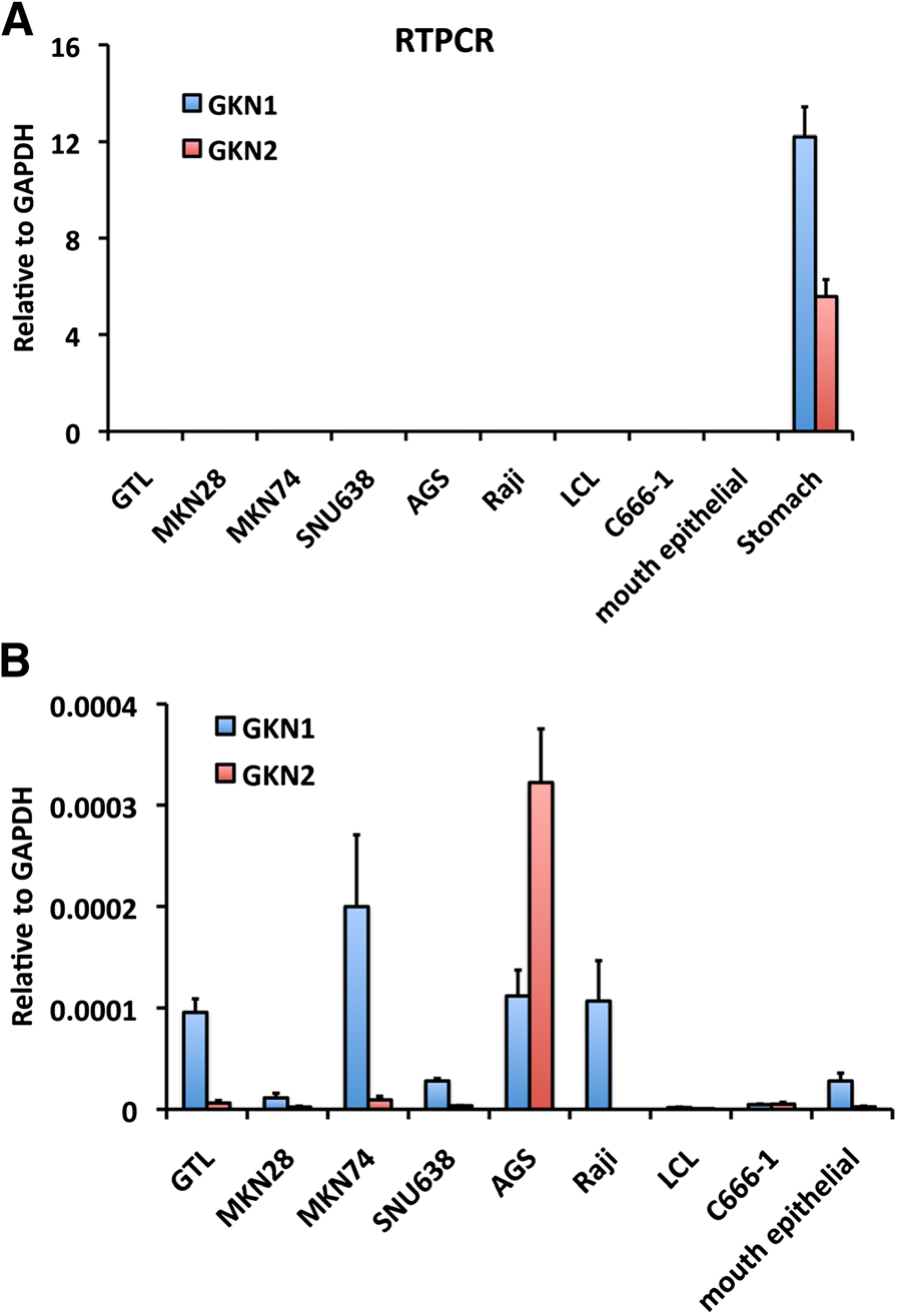
**GKN1 and GKN2 mRNA are highly expressed in primary stomach tissue.** RT-PCR was performed to analyze the mRNA level of GKN1 (blue bars) or GKN2 (red bars) in different cell lines including GC-derived GTL, MKN28, MKN74, SNU638, AGS, BL-derived Raji, EBV immortalized LCL, EBV positive NPC line C666-1, or primary mouth epithelial cells comparing with **(A)** or without **(B)** primary stomach tissue. Error bars indicate standard deviation from the mean (sdm) for n = 3.

We next measured GKN1 or GKN2 mRNA levels in various gastric carcinoma cells lines and in primary normal gastric tissue by qRT-PCR (Figure 2). We found that GKN1 and GKN2 are expressed at much higher levels (~3×10^4^ fold) in primary stomach tissue than in any of the cell lines tested (Figure 2A). Although at much lower levels than primary stomach tissue, GKN1 and GKN2 were both expressed at measurable levels in AGS gastric carcinoma cell lines, with GKN2 expressed at higher levels than all other GC cell lines, or EBV positive B-cell or NPC cell lines (Figure 2B). Very low levels of GKN1 or GKN2 could be detected in primary oral epithelial tissue, further indicating that these genes are specific for primary gastric-tissue.

### EBV latency reduces GKN1 and GKN2 mRNA expression in GC cell lines

**Figure 3:**
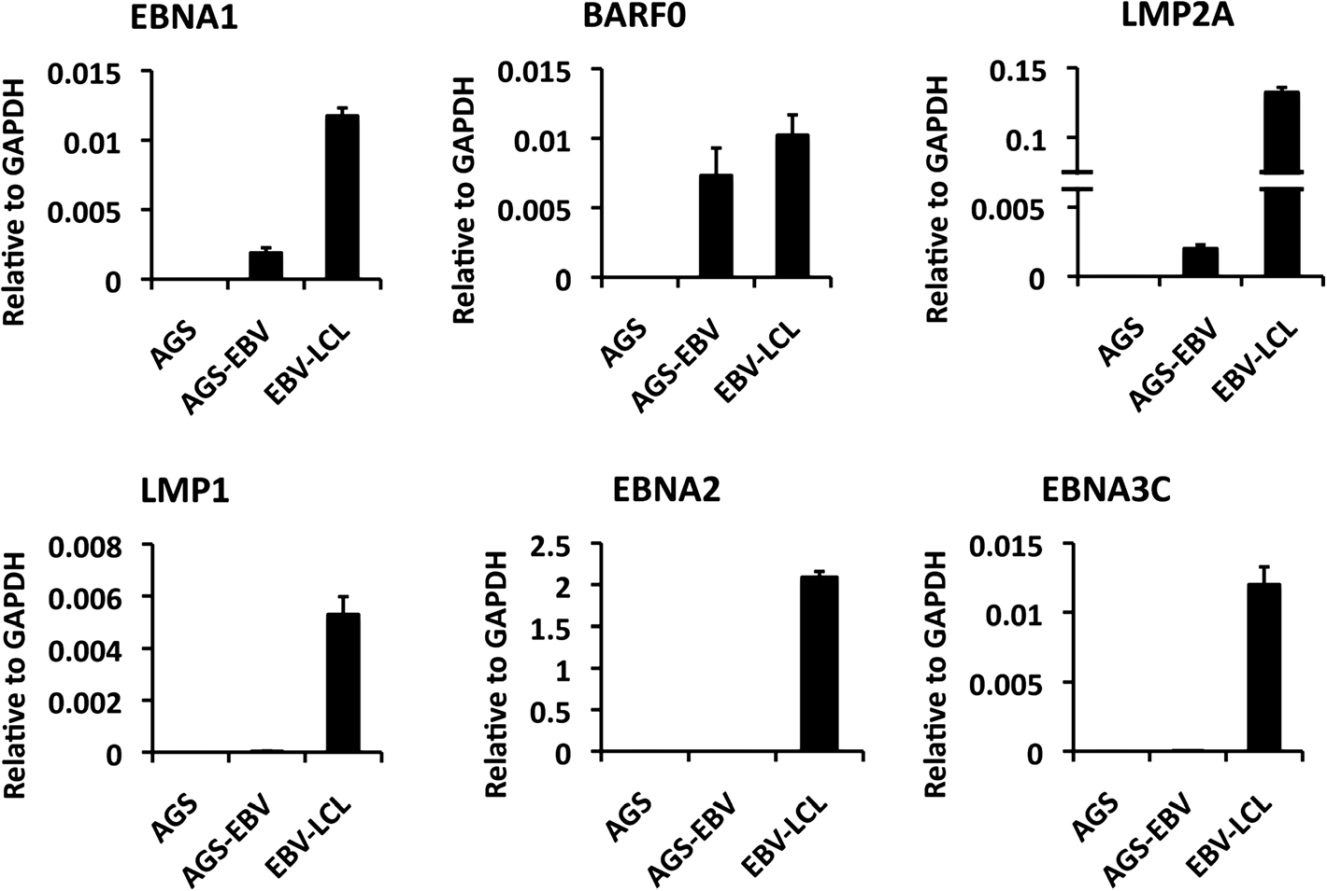
**Characterization of EBV gene expression in AGS-EBV cell lines.** AGS, AGS-EBV positive, and EBV-LCLs were assayed for mRNA expression by qRT-PCR with primers for EBNA1, BARF0, LMP2A, LMP1, EBNA2, or EBNA3C, as indicated. Error bars indicate standard deviation from the mean (sdm) for n = 3.

**Figure 4:**
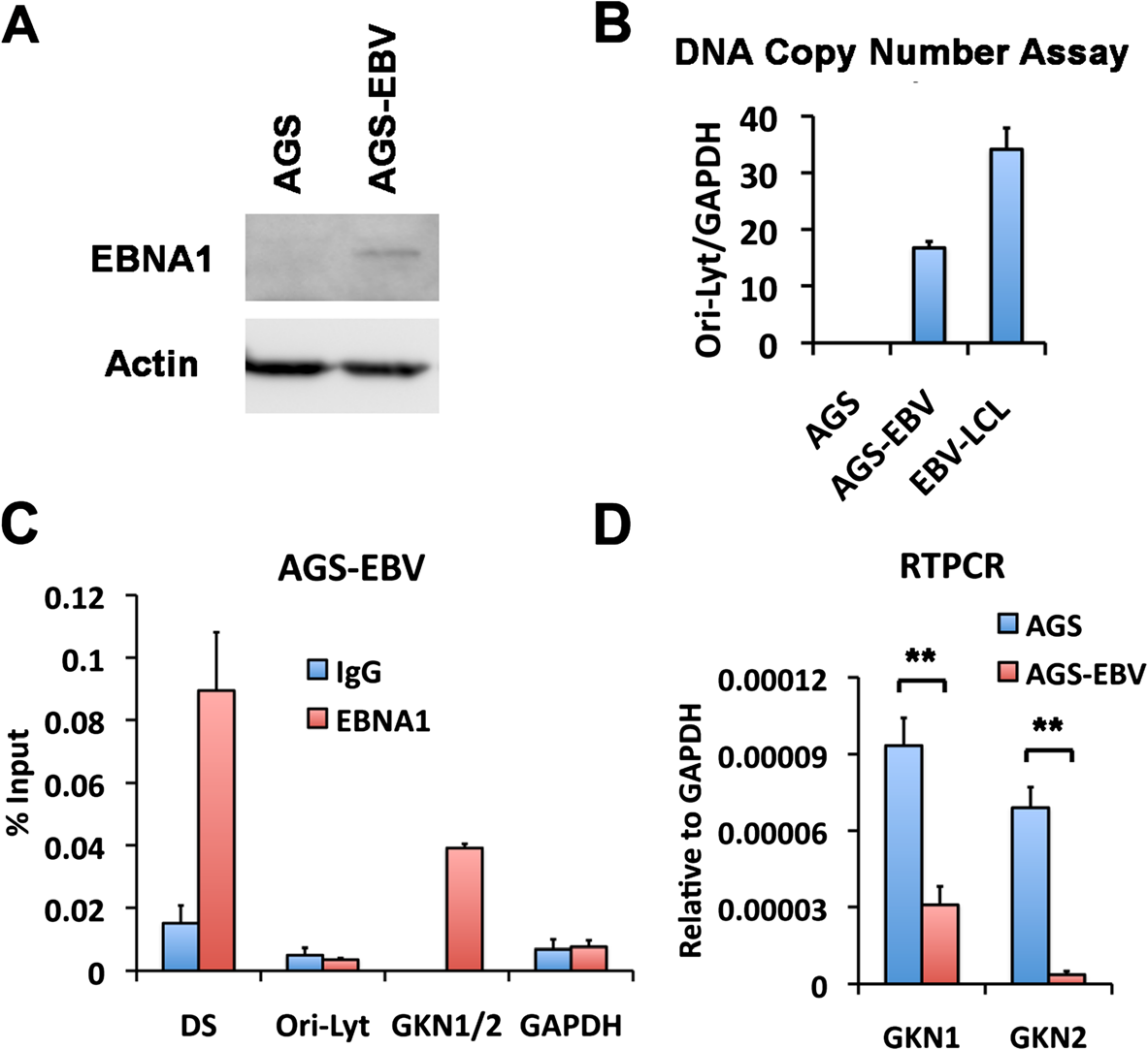
**EBV infection of AGS cells suppresses GKN1 and GKN2 transcription. (A)** Western blot analysis of EBNA1 protein expression in AGS-EBV cells compared with EBV-negative AGS cells was performed using antibody for EBNA1 (top) or Actin (bottom). **(B)** DNA copy number was assayed by real-time PCR of EBV Ori-Lyt DNA relative to the level of cellular GAPDH in AGS, AGS-EBV, and EBV-LCL cells. **(C)** ChIP and real-time PCR analysis of EBNA1 (red bars) or control IgG (blue bars) for DNA binding at the EBV sites including DS and Ori-Lyt, or cellular sites including GKN1/2 and GAPDH in AGS-EBV cells. **(D)** RT-PCR was performed to analyze the mRNA level of GKN1 or GKN2 in AGS (blue bars) or AGS-EBV cells (red bars). ** indicates p < .005. Error bars indicate standard deviation from the mean (sdm) for n = 3.

To test the effect of EBV latent infection on GKN1 and GKN2 expression, we generated an AGS cell line containing EBV B95.8 bacmid. The AGS cell line was selected for hygromycin resistance and GFP positivity to ensure that it contained bacmid components. To characterize the AGS-EBV cell line, we first analyzed the EBV gene expression pattern (Figure 3). We found that AGS-EBV cells expressed detectable mRNA levels of EBNA1, BARF0, and LMP2A, but not LMP1, EBNA2, or EBNA3C (Figure 3). Although EBV B95.8 bacmid lacks the BART miRNAs, these findings are consistent with AGS-EBV cells adopting a variant type I latency gene expression program with some expression of LMP2A, similar to that observed in EBV positive gastric carcinoma tumor tissue [8,10]. To further characterize AGS-EBV cells, we analyzed EBNA1 protein expression by Western blot (Figure 4A) and EBV DNA copy number by qPCR (Figure 4B). EBNA1 was detected as a single low abundance species at the expected molecular mass (Figure 4A). EBV copy number was measured by comparing EBV Ori-Lyt DNA to cellular GAPDH (Figure 4B). We found that AGS-EBV contained ~50% less copies of the viral genome compared to EBV positive LCLs. To determine if EBNA1 retained its DNA binding activity in AGS-EBV, we performed conventional ChIP assays (Figure 4C). We found that EBNA1 bound to the EBV Dyad Symmetry (DS) DNA, as well as to the GKN1-GKN2 binding site in AGS-EBV cells. We next asked whether GKN1 or GKN2 mRNA levels were affected by EBV latent infection in AGS cells by comparing RT-qPCR expression levels in AGS relative to AGS-EBV cells (Figure 4D). We found that GKN1 and GKN2 were repressed ~3-8 fold in AGS-EBV relative to AGS cells, suggesting that EBV latency promotes or stabilizes the transcriptional repression of GKN1 and GKN2.

### EBV increases DNA methylation dependent repression of GKN1 and GKN2 mRNA in GC cell lines

**Figure 5:**
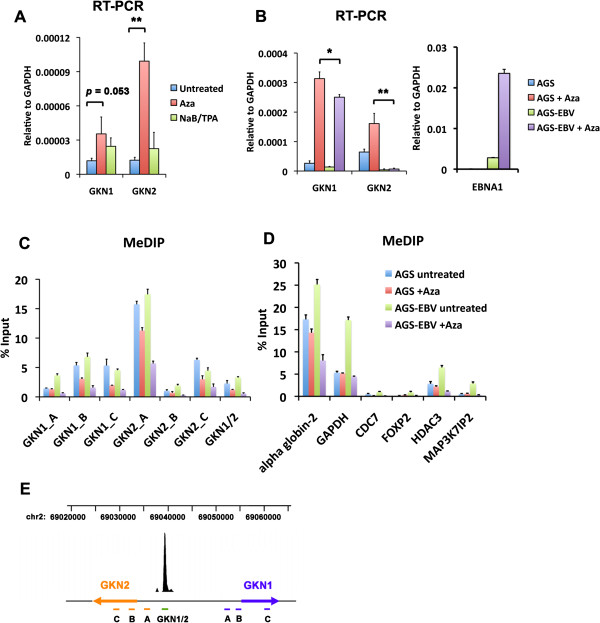
**EBV reinforces the repression of GKN1 and GKN2 gene expression by DNA methylation. (A)** AGS cells were treated with 10 μM Aza, or 1 mM NaB and 20 ng/mL TPA for 48 hours and analyzed by RT-PCR for GKN1 or GKN2 expression level, compared with untreated AGS. **(B)** AGS cells or AGS-EBV cells were treated or untreated with 10 μM Aza for 48 hours, then assayed for GKN1, GKN2 mRNA, or EBNA1 mRNA levels. **(C)** MeDIP assay of AGS or AGS-EBV cells treated or untreated with 10 μM Aza for 48 hours at different DNA regions of GKN1 and GKN2 loci. **(D)** MeDIP assay of AGS or AGS-EBV cells treated or untreated with 10 μM Aza for 48 hrs at cellular regions for alpha globin-2, GAPDH, CDC7, FOXP2, HDAC3, or MAP3KIP2. **(E)** Genome position of primers used for GKN1-GKN2 ChIP and MeDIP assays. * indicates p < .05, ** indicates p < .005. Error bars indicated sdm for n = 3.

GKN1 and GKN2 may be subject to epigenetic suppression in GC and tissue culture cell lines. To explore this possibility, we tested whether treatment with DNA demethylating agent 5′ azacytidine (Aza) or deacetylating agent (NaB) combined with phorbol ester (TPA) would activate GKN1 or GKN2 in AGS cells (Figure 5A). We found that Aza treatment led to a ~10 fold increase in GKN2, and ~ 4 fold increase in GKN1 transcription in AGS treated cells. In contrast, NaB/TPA treatment produced only ~2 fold activation of transcription. These findings suggest that both GKN1 and GKN1 are under active epigenetic repression through DNA methylation. To test if EBV had any effect on Aza induced activation of GKN1 or GKN2, we compared the effects of Aza treatment on AGS relative to AGS-EBV cells by qRT-PCR (Figure 5B). We found that GKN1 and GKN2 were efficiently activated by Aza treatment in AGS cells, but to a lesser extent in AGS-EBV. In these experiments, Aza-induced activation of GKN2 was completely eliminated, while activation of GKN1 was only partly attenuated. Aza-treatment also led to the ~8.4 fold increase in EBNA1 mRNA levels, suggesting that Aza treatment stimulates EBV lytic gene expression in AGS-EBV cell lines. These results suggest the EBV gene products prevent GKN2, and to a lesser extent GKN1 activation after Aza treatment.

To better understand the mechanism of GKN1 and GKN2 epigenetic repression, we examined the DNA methylation levels using methyl cytosine-specific antibody directed DNA immunoprecipitation (MeDIP) assay. We assayed the enrichment of methylated DNA in the GKN1-GKN2 control region in AGS and AGS-EBV cells with or without Aza treatment (Figure 5C). We observed a relative enrichment of methylated CpG at a region between the EBNA1 binding site and GKN2 transcription start site (GKN2_A). Aza treatment led to a decrease in MeDIP signal in most regions where a signal was detected, suggesting that Aza treatment led to a general reduction in DNA methylation. Similar loss of CpG methylation was observed at the alpha-globin gene (which does not bind EBNA1), and at several EBNA1 binding sites, including HDAC3 and MAP3K7IP (Figure 5D). We also noted that MeDIP signals were generally higher in EBV-AGS than in AGS cells, suggesting that EBV latent infection may promote or stabilize DNA methylation throughout the host genome.

### EBNA1 depletion activates GNK1 and GKN2 mRNA expression in EBV positive epithelial cells

**Figure 6:**
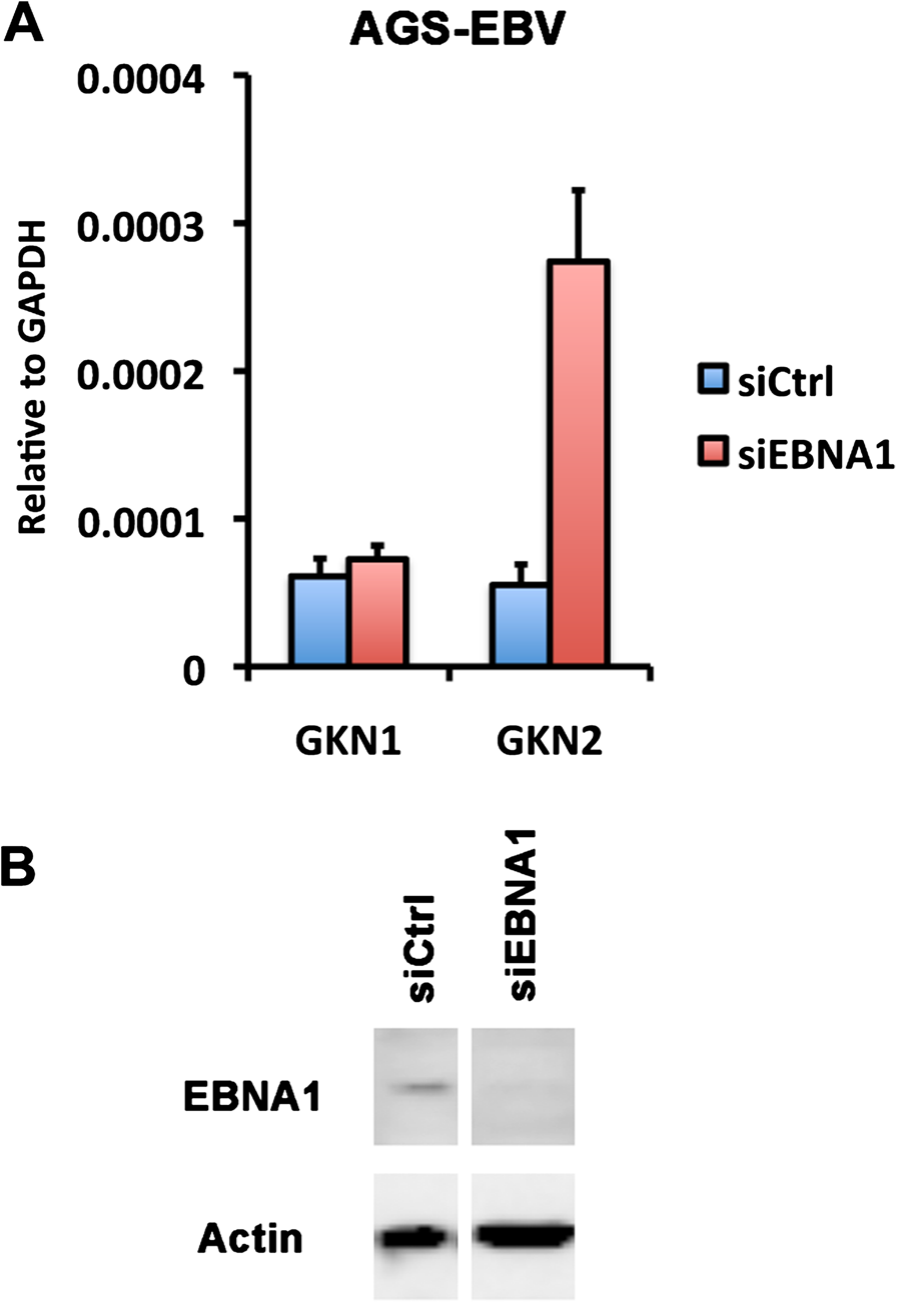
**siRNA depletion of EBNA1 causes de-repression of the GKN1 and GKN2 in AGS-EBV cells.** siCtrl or siEBNA1 transfected AGS-EBV cells were assayed by RT-PCR for GKN1 or GKN2 mRNA level relative to cellular GAPDH **(A)**. Cells were harvested at 72 hours post-transfection with siRNA. Western blot showing EBNA1 (top panel) and loading control Actin (lower panel) in AGS-EBV cells **(B)**. Error bars indicate standard deviation from the mean (sdm) for n = 3.

To determine if EBNA1 contributed to the transcriptional repression of GKN1 and GKN2, we first attempted to deplete EBNA1 in AGS-EBV cells using siRNA (Figure 6). We generated a siRNA targeting the 3′ non-coding UTR of EBNA1 mRNA, which partially depleted EBNA1 protein in AGS-EBV cells (Figure 6B). Depletion of EBNA1 resulted in an ~5 fold activation of GKN2, with little detectable activation of GKN1 (Figure 6A). These findings suggest that EBNA1 may function as a transcriptional repressor of GKN2 in AGS-EBV cells.

### EBNA1 inhibits GKN1 and GKN2 transcription after DNA demethylation

**Figure 7:**
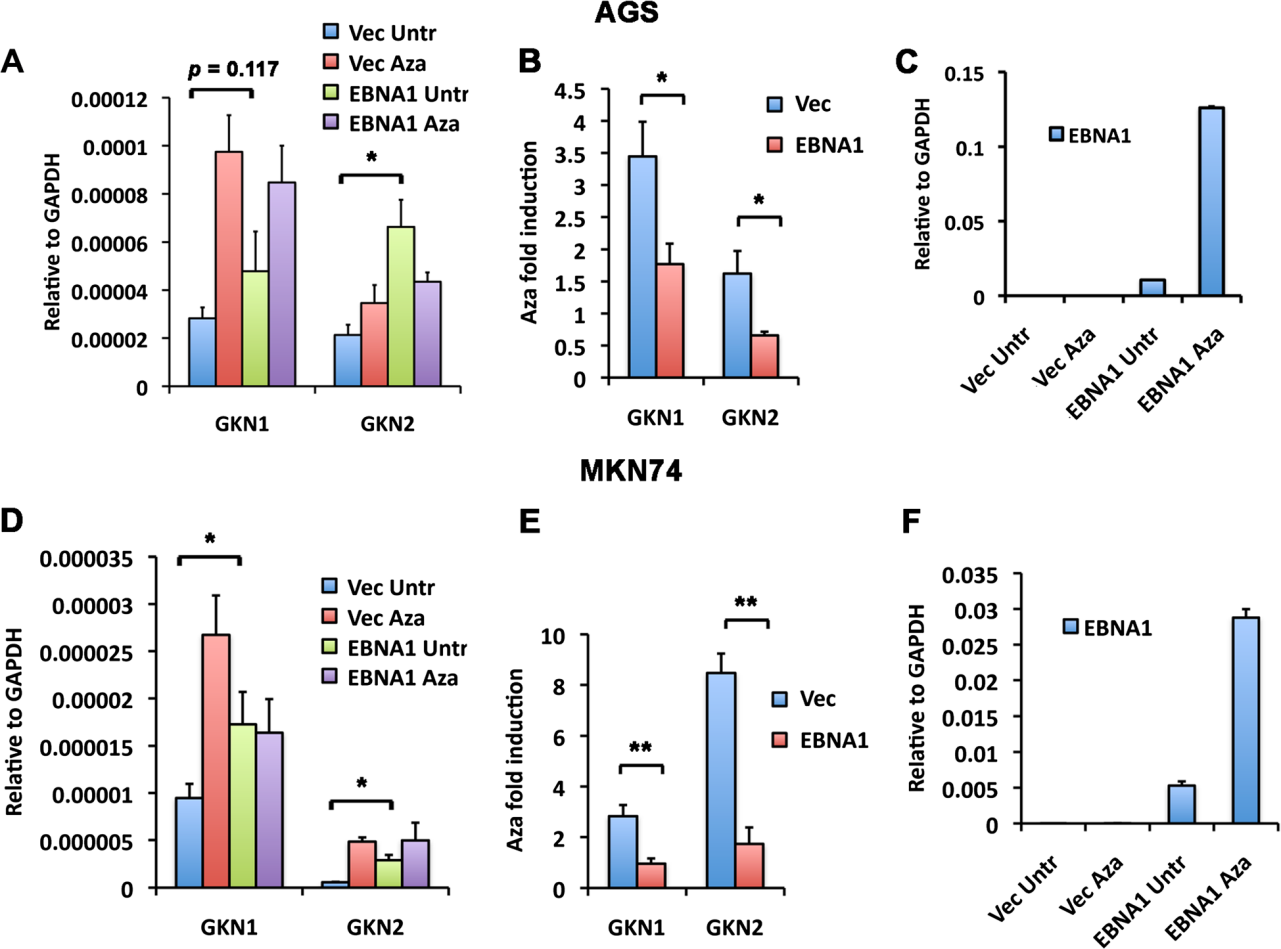
**EBNA1 inhibits GKN1 and GKN2 transcription after DNA demethylation in GC cells. (A)** AGS cells were transduced with pLU-Vector (Vec) or pLU-EBNA1 and then untreated (Untr) or treated with 10 μM 5′-azacytidine (Aza) for 48 hrs. GKN1 and GKN2 mRNA were quantified by qRT-PCR relative to GAPDH. **(B)** Fold induction of GKN1 and GKN2 mRNA by Aza were quantified from panel **A**. **(C)** qRT-PCR analysis of EBNA1 mRNA levels in AGS cells. **(D)** Same as in **A**, except for using MKN74 rather than AGS cells. **(E)** Fold induction of GKN1 and GKN2 mRNA quantified from panel **D**. **(F)** qRT-PCR analysis of EBNA1 mRNA levels in MKN74 cells. *indicates p < .05, **indicates p < .005. Error bars indicated sdm for n = 3.

To further explore the contribution of EBNA1 to GKN1 and GKN2 transcription regulation, we tested the effect of ectopic expression of EBNA1 alone on Aza-induced levels of GKN1 or GKN2 transcription in GC cells. AGS cells were transduced with an EBNA1 expressing lentivirus. Stable AGS-EBNA1 (pLU-EBNA1) or AGS- control vector (pLU-Vec) cell lines were selected and assayed for GKN1 and GKN2 mRNA levels. We observed that AGS-EBNA1 cells had a ~2-3 fold higher basal level of GKN1 and GKN2 mRNA relative to parental AGS cells (Figure 7A). However, Aza-induced mRNA levels of GKN1 and GKN2 were attenuated in AGS-EBNA1 compared to AGS cells (Figure 7B). Aza treatment also led to a large increase in EBNA1 mRNA levels (Figure 7C). To determine if the effects of EBNA1 on GKN1 and GKN2 were specific to AGS cells, we transduced another EBV negative GC cell line MKN74 with EBNA1 lentivirus (Figure 7D-F). Similar to AGS cells, we found that EBNA1 increased basal levels of GKN1 and GKN2, but inhibited the ability of Aza to further induce GKN1 and GKN2 mRNA levels (Figure 7D and E). We confirmed that Aza-treatment was effective by measuring EBNA1 mRNA levels, which increased ~7 fold (Figure 7F). These findings suggest that ectopic expression of EBNA1 can increase basal, but inhibit Aza-induced levels of GKN1 and GKN2 transcription in EBV-negative GC cell lines.

## Discussion

In this study, we identified a high-occupancy EBNA1 binding site in the 5′ promoter control region of the divergently transcribed GKN1 and GKN2 genes. EBNA1 binding sites were observed in two independent ChIP-Seq data sets from EBV positive lymphoid BL cells Raji and EBV positive epithelial nasopharyngeal carcinoma cells C666-1 (Figure 1A). We confirmed these binding sites by conventional ChIP–qPCR in both cell lines (Figure 1B and C). EBNA1 was also shown to bind directly to these sites by EMSA with purified recombinant EBNA1 DBD protein (Figure 1D). We show that GKN1 and GKN2 mRNA levels are highly repressed in most cell lines relative to primary gastric tissue (Figure 2). To study the potential role of EBV and EBNA1 in the transcriptional control of GKN1 and GKN2, we generated an EBV positive AGS gastric carcinoma cell line. We show that EBV adopts a variant type I latency pattern in AGS cells (Figure 3), and that EBNA1 can bind to the GKN1/GKN2 promoter region in the cellular chromosome (Figure 4C). We also found that GKN1 and GKN2 mRNA were further suppressed in EBV positive AGS cells relative to control EBV negative AGS cells (Figure 4D). We then showed that Aza-treatment led to the increase expression of GKN1 and GKN2 (Figure 5A), and that EBV latent infection inhibits Aza activation of GKN2 (Figure 5B). We found that siRNA depletion of EBNA1 in EBV positive AGS cells leads to transcription activation of GKN2 (Figure 6). We also show that EBNA1 ectopic expression moderately increases basal, but inhibits the Aza-induced levels of GKN1 and GKN2 transcription (Figure 7). Taken together, these findings indicate that EBNA1 binds to the GKN1-GKN2 promoter control region in multiple cell types, and raise the possibility that EBNA1 contributes to the transcriptional and epigenetic repression of the GKN1 and GKN2 tumor suppressor genes in EBV positive GC.

EBV latent infection is known to increase the tumorigenic phenotype of gastric carcinoma cells [29-31]. GKN1 and GKN2 are reported to function as cell growth inhibitors and tumor suppressors in GC [20,21,23,25-27]. Our mRNA expression data showing high-level mRNA expression only in primary normal gastric tissue are consistent with a role of GKN1 and GKN2 as a tumor suppressor. However, we were unable to show that over-expression of either or both GKN1 or GKN2 in AGS or AGS-EBV cause a cell cycle arrest or reduce viability (data not shown). This suggests that GKN1 and GKN2 function at earlier stages in tumor cell evolution, or in more complex tumor microenvironments. We speculate that EBNA1 may have a more pronounced effect on GKN1 and GKN2 expression in situations where EBV may infect primary gastric cells where basal expression of GKN1 and GKN2 are high and important for tumor suppression.

Previous published studies have shown that GKN1 and GKN2 transcription is subject to epigenetic suppression by DNA methylation in all forms of GC [21]. Our studies are consistent with the role of DNA methylation in the epigenetic suppression of GKN1 and GKN2 in AGS cells. Treatment with Aza resulted in the 4-10 fold increase in GKN1 and GKN2 mRNA expression (Figure 5A), and MeDIP revealed enrichment of methylated DNA at the promoter regions (Figure 5C). AGS-EBV cells did show an increase in DNA methylation at several cellular sites, including regions surrounding the EBNA1 binding sites at the GKN1 promoter region (Figure 5C), and the HDAC3 and MAP3K7IP2 genes (Figure 5D). However, the presence of EBNA1 in AGS-EBV cells did not prevent Aza-induced demethylation at these sites. This suggests that EBNA1 may repress transcription from some promoters, like GKN2, through a mechanism distinct from DNA methylation. However, ectopic expression of EBNA1 alone produced a more complicated phenotype, causing a small increase in basal expression, but limiting the effects of Aza-induced demethylation (Figure 7). This may suggest that that EBNA1 may function differently when expressed ectopically, than when expressed in the context of the viral genome. Nevertheless, our findings suggest that EBNA1 perturbs the normal transcriptional regulation of the GKN1 and GKN2 genes.

The precise function of EBNA1 in transcription regulation remains unclear. EBNA1 has been implicated in the transcriptional activation and repression of both viral and cellular genes [32,33]. EBNA1 can repress its own mRNA expression from the EBV Qp in type III latency, where repression has been linked to steric interference with RNA polymerase II binding to the transcription initiation site [34]. On the other hand, EBNA1 can activate Cp and LMP1 promoters in type III latency where it may function as an enhancer-like factor [35-37]. EBNA1 has been implicated in transcription activation of some cellular genes, including the Nox2 gene involved in reactive oxygen species formation [19]. EBNA1 may also affect host-cell transcription through a global remodeling of the host chromosome [38]. Thus, EBNA1 may alter cellular transcription through multiple direct and indirect mechanisms.

Epigenetic modifications are known to play an important role in EBV-associated gastric carcinoma [39]. Interestingly, AGS cells carrying EBV bacmid genomes had higher levels of methylated DNA at many tested sites (Figure 5D). This is consistent with the proposed role of EBV in the methylation of host tumor suppressor genes [40]. This is also consistent with the findings that EBV positive GC has elevated DNA methylation at promoter regions of several key GC tumor suppressors, including gastrokine genes [39,41-45]. While EBNA1 bound near DNA methylated regions of the GKN2, we were unable to show that EBNA1 modulates DNA methylation at the GKN1 and GKN2 sites (data not shown). However, it is possible that EBNA1 in association with another viral encoded or induced factor may stabilize GKN1 and GKN2 transcriptional repression through a chromatin-dependent and structural mechanism that reinforces DNA methylation. It is also possible that EBNA1 may regulate GKN1 or GNK2 only in tissue or tumor microenvironments that are not readily recapitulated in cell culture. While the function of EBNA1 binding to host cell chromosome sites remains an important area of investigation, more sophisticated infection models may be required to elucidate its potential role in altering host cell gene expression and carcinogenesis.

## Methods

### Cells, plasmids, and lentivirus infection

EBV-positive Burkitt’s lymphoma Raji cells, EBV positive nasopharyngeal carcinoma C666-1 cells, and gastric carcinoma cell lines (a gift from Dr. Antonia R. Sepulveda, Columbia University) including GTL, MKN28, MKN74, SNU638 were maintained in RPMI containing 10% FBS and supplemented with antibiotics (penicillin and streptomycin). Gastric carcinoma AGS cells (ATCC No. CRL-1739) were maintained in F-12K containing 10% FBS. Primary mouth epithelial cells were provided by Dr. Manjunatha Benakanakere, University of Pennsylvania and cultured in Keratinocyte-SFM medium. EBV-LCL was established by primary infection of peripheral blood mononuclear cells (PBMC) with EBV BAC virions generated from stimulated 293-EBV cells [46,47]. EBV-LCL contains a hygromycin B resistant EBV bacmid were maintained in RPMI containing 10% FBS, hygromycin B (100 μg/ml), glutamax (Invitrogen), and antibiotics. AGS-EBV cells were generated from AGS cells co-cultivated with EBV-LCL by adapting a previously published co-cultivation method described AGS cells infection with rEBV through cell-to-cell contact [48] with some modification. Briefly, EBV-LCL was induced by 20 ng/mL 12-*O*-tetradecanoylphorbol-13-acetate (TPA) and 1 mM sodium butyrate (NaB) 24 hours prior to co-cultivation. The induced EBV-LCL cells were washed with PBS twice to completely remove the inducing agents, resuspended with complete RPMI medium at 10^6^ cells/ml before the co-incubation. AGS cells were plated in 6-well Plates 24 hours before co-cultivation, then 60 - 70% confluent AGS cells in 1 ml complete F-12K medium were incubated with 10^6^ induced and washed EBV-LCL cells in 1 ml complete RPMI. 24 hours later, 2 ml of Serum free F-12K medium was added to each well to reduce the FBS concentration to 5% to prevent cell overgrowth. 3 days after co-incubation, EBV-LCL cells were removed from the co-cultures and the AGS cells were thoroughly washed with PBS at least 5 times to remove any of the donor EBV-LCL cells. The infected AGS cells was then incubated with fresh F-12K medium with 10% FBS and 100 μg/ml hygromycin B and the selection medium was changed every 2 to 3 days until the infected AGS cells formed Hyg B selection colonies with GFP expression (usually 3 to 4 weeks after co-cultivation). The selection colonies were then pooled and tested for EBNA1 expression and EBV genome copy number before subject to experiments. AGS-EBV cells were maintained in F-12K medium with 10% FBS and 100 μg/ml hygromycin B.

pLU-EBNA1 Lentivirus expression vector was constructed by PCR amplification of EBNA1 with primers (GCGGGATCCTCTGACGAGGGGCCAGGTACAGGACCT and ATCGTCGACTCACTCCTGCCCTTCCTCACCCTCATC) introducing a 5′ BamH I and 3′ Sal I site cloned in frame to pLU-TCMV-FMCS-pPURO. AGS or MKN74 cells were infected with Lentivirus expressing pLU-EBNA1 or pLU control vector generated freshly from 293T cells. Infected AGS or MKN74 cells were then selected with 2.5 μg/ml Puromycin for 10 to 14 days. The selected cells were pooled and treated with or without 5'-azacytidine for 48 hours then subjected to RT-PCR.

siRNA against EBNA1 and siRNA Control (Cat. No. D-001810-01-20) were all purchased from Dharmacon. siRNA directed against EBNA1 3′UTR were synthesized using the target sequence CGGAGAUGACGGAGAUGAAUU. Transfection of siRNA duplexes was conducted by using Oligofectamine (Dharmacon), following manufacturers specifications.

### Chromatin immunoprecipitation (ChIP) assays

ChIP assays were performed as described previously [49]. Quantification of precipitated DNA was determined using real-time PCR and the delta Ct method for relative quantification (ABI 7900HT Fast Real-Time PCR System; Applied Biosystems). Primers for ChIP assays are available upon request. The following antibodies were used for ChIP assays: rabbit anti-EBNA1 (305/10 wk), anti- rabbit IgG (Santa Cruz Biotechnology, sc-2027). Anti-Actin HRP (Sigma, A3854) and rabbit anti-EBNA1 (305/10 wk) were used for Western Blotting. EBNA1 specific antibody was raised against a bacterial EBNA1 protein lacking the GA-repeat region using New Zealand white rabbits and then affinity purified using the same bacterial protein coupled to sepharose beads.

### EMSA

DNA fragments were labeled using T4 polynucleotide kinase (NEB) in the presence of [γ-^32^p]ATP and purified using G-25 spin columns (GE Healthcare). Purified EBNA1 DNA binding domain [17] was incubated with probes at room temperature for 30 min in a total volume of 20 μl buffer containing 10mM TrisCl PH 8.0, 100 mM KCl, 1 mM EDTA, 10 mM MgCl_2_, 0.05 μg/μl poly(dI-dC), 0.5 μg/μl bovine serum albumin, 0.05% NP40, 35 mM *β*-mercaptoethanol, and 10% glycerol. The samples were then separated by electrophoresis on a native 5% polyacrymide gel. Gels were dried and analyzed using a Typhoon phosphorImager system. The forward probe sequences are gatccggatacagattaggatagcatatactaccca (FR), tttcttattttccaggcagcatatactatctcaagctcaccatgtc (GKN1/2) and cgccgccggggcctgcggcgcctcccgcccgggcatggggccgcgc (negative control, LANA binding sites at KSHV TR).

### Quantitative RT-PCR

For cell lines, RNA was isolated from 2 × 10^6^ cells using RNeasy Kit (Qiagen) and then further treated with DNase I. Reverse transcriptase PCR (RT-PCR) was performed as previously described [50]. Real-time PCR was performed with SYBR green probe in an ABI Prism 7900 according to the manufacturer’s specified parameters. Primer sequences for RT-PCR are available upon request. Total human stomach RNA (Cat. No. 540037) was purchased from Agilent Technologies.

### Genome copy number assay

Genome copy number assay was performed as described previously [51]. The total cellular DNA was assayed by real-time PCR using primers for the EBV Ori-Lyt region and normalized by the cellular GAPDH DNA region. The primers for Ori-Lyt and GAPDH are available upon request.

### MeDIP

Methyl-DNA immunoprecipitation (MeDIP) methods have been described previously [51]. MeDIP DNA were analyzed by real-time PCR with delta Ct method for quantification. The primers for MeDIP are available upon request.

## Competing interests

The authors declare no competing interests, with the exception that PML declares an interest in Vironika, LLC that is developing small molecule inhibitors for EBNA1.

## Author contributions

FL and PML conceived of the study, developed its design, and drafted the manuscript. FL carried out the molecular genetic studies. IT participated in ChIP-seq studies. HTL participated in cell-based assays. KD carried out RTPCR. All authors read and approved the final manuscript.
